# Combined Catheter Ablation and Left Atrial Appendage Occlusion in Atrial Fibrillation: From Data to Clinical Reality

**DOI:** 10.1007/s10557-025-07685-2

**Published:** 2025-04-16

**Authors:** Kyriakos Dimitriadis, Eleni Adamopoulou, Nikolaos Pyrpyris, Panagiotis Iliakis, Eirini Beneki, Dimitrios Konstantinidis, Christos Fragkoulis, Alexios Antonopoulos, Aggelos Papanikolaou, Konstantinos Aznaouridis, Konstantina Aggeli, Konstantinos Tsioufis

**Affiliations:** First Department of Cardiology, School of Medicine, National and Kapodistrian University of Athens, Hippokration General Hospital, Vas Sofias 114, 115 27 Athens, Greece

**Keywords:** Atrial fibrillation, Stroke, Left atrial appendage occlusion, Ablation, Anticoagulation, Thromboembolism

## Abstract

**Purpose:**

Atrial fibrillation (AF) requires treatment that focuses on two main goals: symptom control and prevention of thromboembolic events. Catheter ablation and left atrial appendage occlusion (LAAO) constitute two well-established treatment methods in selected patients that accomplish these two goals correspondingly. Recently, there is increasing interest in performing the two procedures concomitantly in a so-called “combined” or “one-stop” procedure. This review aims to summarize the current data on the combined procedure, from the rationale and the techniques to its clinical efficacy, indications and future directions.

**Methods:**

An extensive search has been conducted using the MEDLINE/PubMed database to identify the relevant studies.

**Results:**

The reported success rates of the combined procedure are very high and frequently reach 100% when performed by experienced operators. The periprocedural and follow-up complications are low, the procedure is cost-effective, while there is significant stroke, bleeding and arrhythmia incidence reduction that does not seem to be undermined by interference between the two interventions. There are also a few indications that the one-stop procedure has a positive effect on left atrial mechanical function as it has been correlated with left atrial reverse remodeling. On the other hand, some studies suggest possible increase in peri-device leaks (PDLs), compared with LAAO alone, which could in turn negatively affect the clinical outcomes. Most available studies are small and observational, with a lack of randomized controlled trials.

**Conclusion:**

Catheter ablation and left atrial appendage occlusion can be safely and effectively combined in a cost-effective single procedure in carefully selected patients.

**Graphical Abstract:**

AF = Atrial Fibrillation, CA = Catheter Ablation, DAPT = Dual Antiplatelet Therapy, LA = Left Atrium, LAAO = Left Atrial Appendage Occlusion, OAC = Oral Anticoagulation, PDL = Peri-device Leakage, RF = Radiofrequency

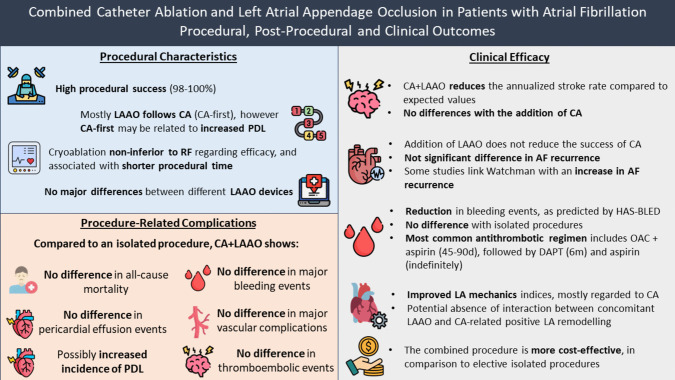

## Introduction

Atrial fibrillation (AF), the most common cardiac arrhythmia, is well-known for its threatening complications, with the most significant being ischemic stroke. While the stroke risk is usually managed with oral anticoagulant treatment (OAC) according to the CHA_2_DS_2_-VASc score, this is not applicable to all cases. Some patients may have contraindications for long-term anticoagulation or significantly elevated bleeding risk, while others may have experienced a stroke under OAC or even be unwilling to take the medication. In these cases, considering that most of the thrombus formations occur in the left atrial appendage (LAA), occlusion of LAA (LAAO) can be a suitable alternative as suggested by the latest clinical practice guidelines of ESC [1] and the EHRA/EAPCI expert consensus statement [2].

Catheter-based AF ablation (CA) is a well-established treatment for the prevention of AF recurrence and is thought not only to be safe, but also superior to antiarrhythmic drugs in terms of sinus rhythm (SR) maintenance and symptom control. However, no randomized trial has yet managed to show a significant reduction in all-cause mortality, stroke or major bleeding. The current guidelines therefore propose indefinite OAC continuation in patients subjected to CA with high stroke risk independent of the rhythm status [1].

In this context and given the shared techniques of these interventions, research is focusing on the feasibility and value of a combined procedure consisting of both CA and LAAO, which offers concomitant rhythm control and stroke prevention therapy in carefully selected patients.

## Procedure Techniques and Success Rates

### Procedure Sequence

The two interventions share common techniques, namely groin vein access, septal puncture and left atrium manipulation. Nonetheless, when performed simultaneously, they could importantly interfere with each other, possibly undermining their success. When CA is performed first, the applied radiofrequency heating or cryoballoon freezing causes acute tissue edema, which could result in improper LAAO device size selection and subsequent peri-device leak (PDL). Indeed, the left atrial ridge (LAR) has been found significantly increased after the CA procedure [3, 4]. Moreover, as the ideal septal puncture site vaguely differs between the two interventions, with LAAO puncture site ideally being lower than CA, manipulation and apposition difficulties may occur [5]. Lastly, use of transesophageal echocardiography (TOE) for LAAO guidance is more likely to cause esophageal injuries when the surrounding endocardium has already endured heat-induced lesions during CA [6].

Research, however, has demonstrated remarkable success rates of this sequence. Available studies and their characteristics, as well as related outcomes are reported in Tables 1, 2, 3 and 4. The EWOLUTION and WASP registries showed that all 139 combined procedures were successful [7], while a more extensive subsequent analysis demonstrating a 99.3% success rate [8]. Another registry of 349 patients reports a 100% successful sealing, defined as no flow or a remaining jet < 5 mm at the end of the procedure [9]. Almost all the currently available prospective observational studies report a 100% success rate [10–15], with only one exception where the rate was 97% [16]. Similarly, the retrospective studies report a 100% success rate [4, 6, 17–29], except for the CLACBAC study which reports a 97.4% [30] and 97.6% [31] rate, as well as Preisendörfer et al. [32] where the rate was 95.8%. Thus, the success rate of LAAO after CA is high and even similar to isolated LAAO [33, 34].

**Table 1:** Registries investigating combined catheter ablation and left atrial appendage occlusion

Author	Number of patients	Age	CHA_2_DS_2_-VASc	HAS-BLED	Ablation energy	LAAO Device	FU time	Procedural success
Chen et al. 2023 [35]	529 men	68.7 ± 8.1	3.1 ± 1.5	2.4 ± 1.0	Radiofrequency	Watchman	22.9 (11.6 ± 33.8) months	N/A
	402 women	71.3 ± 7.4	4.1 ± 1.5	2.3 ± 1.0	Radiofrequency	Watchman		N/A
	*p*	*p* < 0.001	*p* < 0.001	*p* = 0.140	Radiofrequency	Watchman		N/A
Liu et al. [36]	50	64.9 ± 7.7	3.7 ± 1.4	2.5 ± 0.9	40 radiofrequency, 10 cryoballoon	48 Watchman, 2 Amplatzer Cardiac Plug	20.2 ± 11.5 months	N/A
Phillips et al. 2018 [7]	139	64.1 ± 7.3	3.4 ± 1.4	1.5 ± 0.9	105 irrigated radiofrequency, 1 cryoballoon, 29 non-irrigated phased radiofrequency multielectrode, 4 non-recorded	Watchman	1 month	100%
Phillips et al. 2019 [8]	142	64.2 ± 7.2	3.4 ± 1.4	1.5 ± 0.9	106 irrigated radiofrequency, 1 cryoballoon, 30 non-irrigated phased radiofrequency multielectrode, 5 non-recorded	Watchman	726 ± 91 days	99.3%
Wintgens et al. [9]	349	63.1 ± 8.2	3.0 (2.0 – 4.0)	3.0 (2.0 – 3.0)	79% irrigated radiofrequency, 21% phased radiofrequency	Watchman	34,5 (24–44) months	100%

Author	Νο Residual leak or leak ≤ 5 mm	Total adverse events	Stroke by the end of FU time	Major bleeding events by the end of FU time	AF Recurrence	DRT	Antithrombotic treatment after the procedure	Freedom from OAC by the end of FU time
Chen et al. 2023 [35]	14.3% after 3 months	2.1% major, 1.3% minor complications	0.4% (Stroke/TIA/SE composite)	0.6%	N/A	1.6%	N/A	N/A
	26.0% after 3 months	2.2% major, 3.7% minor complications	0.5% (Stroke/TIA/SE composite)	0.2%	N/A	1.6%	N/A	N/A
	*p* = 0.112	*p* = 0.868 (major complications) *p* = 0.027 (minor complications)	*p* > 0.999	*p* = 0.462	p non-significant	*p* > 0.999	N/A	N/A
Liu et al. [36]	100% after 6 months	8% periprocedural complications, 6% during follow-up	2%	0%	36%	0%	N/A	N/A
Phillips et al. 2018 [7]	100% after the procedure, 98,1% by the end of the FU time	8,7% by the end of FU time	0	2,9%	N/A	2.1%	92.8% OAC, 5.8% anti-platelet, 1.4% no therapy	N/A
Phillips et al. 2019 [8]	100% after the procedure	N/A	0.7%, 0.36 per 100 patient-years	3.52%, 1.83 per 100 patient-years	N/A	2.1%	92.9% OAC, 5.6% anti-platelet, 1.4% no therapy	92%
Wintgens et al. [9]	100% after the procedure, 98.9% in first TOE follow-up within 3 months	7.2% up to 30 days after the procedure	2%	3.2%	51%	1.1%	N/A	84.9%

**Table 2:** Randomized controlled trials and Prospective observational studies investigating combined catheter ablation and left atrial appendage occlusion

Author	Number of patients	Age	CHA_2_DS_2_-VASc	HAS-BLED	Ablation energy	LAAO Device	FU time	Procedural success
Alipour et al. [10]	62	64 ± 8	3.0 (2.75 – 4.00)	2.0 (2.0 – 3.0)	Radiofrequency	Watchman	38 (25 – 45) months	100%
Calvo et al. [16]	35	70 ± 7	3.1 ± 1.1	3.1 ± 1	Radiofrequency	Watchman (29 patients) Amplatzer Cardiac Plug (6 patients)	13 (3–75) months	97%
Chen et al. 2020 [12]	178	68.9 ± 8.1	3.3 ± 1.5	1.6 ± 1.0	N/A	Watchman	Up to one year	100%
Chen et al. 2021 [15]	56	69.4 ± 7.5	4.0 (3.0–5.0)	3.0 (2.0–3.0)	Radiofrequency	LAmbre	12 months	100%
Fassini et al. [11]	35	72 ± 4	100% ≥ 2	37% ≥ 2	Cryoballoon	Watchman (10 patients) Amplatzer Cardiac Plug (25 patients)	24 ± 12 months	100%
Lakkireddy et al. [37]	Combined group: 378	66.2 ± 8.42	1.6 ± 1.10	N/A	Radiofrequency	LARIAT	12 months	94% (for CA)
	CA-only group: 198 *Modified intention to treat population	67.4 ± 7.45	1.6 ± 1.17	N/A	Radiofrequency	N/A	12 months	94% (for CA)
	p	N/A	N/A	N/A	N/A	N/A	N/A	N/A
Romanov et al. (RCT) [38]	Combined group: 45 (intention-to-treat), 39 (on-treatment)	60 ± 5	2.2 ± 0.6	3.5 ± 0.8	Radiofrequency	Watchman	24 months	87%
	Catheter ablation – only group: 44 (intention-to-treat), 50 (on-treatment)	60 ± 6	2.3 ± 0.7	3.4 ± 0.8	Radiofrequency	N/A	24 months	100%
	*p*	*p* = 0.82	*p* = 0.84	*p* = 0.66	N/A	N/A	N/A	N/A
Sun et al. [39] A	HFrEF: 20 patients	67.3 ± 9.8	3.6 ± 1.3	2.7 ± 1.3	Radiofrequency	Watchman	27.4 ± 7.5 months	100% (for CA)
	HFmrEF: 45 patients	67.3 ± 6.4	4.0 ± 1.1	2.8 ± 1.0	Radiofrequency	Watchman		100% (for CA)
	*p*	*p* = 0.967	*p* = 0.225	*p* = 0.848	N/A	N/A		*p* > 0.999
Sun et al. [39] B	Combined procedure (HFrEF, HFmrEF): 65 patients	67.3 ± 7.5	3.9 ± 1.2	2.7 ± 1.0	Radiofrequency	Watchman	27.4 ± 7.5 months	100% for CA
	No procedure: 138 patients	69.2 ± 9.9	4.2 ± 1.5	2.8 ± 0.9	N/A	N/A		N/A
	*p*	*p* = 0.172	*p* = 0.159	*p* = 0.477	N/A	N/A		N/A
Swaans et al. [13]	30	62.8 ± 8.5	3 (3–5)	2 (1–3)	Radiofrequency	Watchman	12 months	100%
Zhao et al. [14]	84	68.7 ± 8.0	3.4 ± 1.4	2.2 ± 1.2	Radiofrequency	Watchman	6 months	100%

Author	Νο Residual leak or leak ≤ 5 mm	Total adverse events	Thromboembolic events by the end of FU time	Major bleeding events	AF recurrence	DRT	Antithrombotic treatment after the procedure	Freedom from OAC by the end of FU time
Alipour et al. [10]	100% after the procedure, 95% after 60 days	8% minor periprocedural complications, 0% major periprocedural complications	4,8% strokes (1.7% annual stroke risk, 74% fewer strokes)	4.8% during follow-up	42%	0% after 60 days	N/A	78%
Calvo et al. [16]	100% after 3 months	8.6% periprocedural complications, 5.7% follow-up complications	2.86% (TIA)	2.86% (haemorrhagic stroke)	22%	0% after 3 months	8.6% OAC, 74% OAC + aspirin, 8.6% LMWH, 8.6% LMWH + aspirin	97%
Chen et al. 2020 [12]	100% after the procedure, 100% at 3 months	N/A	1.2% stroke	0% until 3 months, 1,4% after one year	14% after 3 months, 37.8% after 1 year	0% at 3 months	67.4% warfarin, 19.7% dabigatran, 12.9% rivaroxaban, 4.5% additional antiplatelet therapy	N/A
Chen et al. 2021 [15]	100% after the procedure and after 12 months	3.6% periprocedural complications	0%	1.8%	25%	4%	73.2% Rivaroxaban, 17.8% Dabigatran, 9.0% Warfarin	96.4%
Fassini et al. [11]	100% after the procedure, 100% at first TOE follow-up (30–60 days after)	3% periprocedural complications	0%	N/A	29%	0%	N/A	86%
Lakkireddy et al. [37]	99% after the procedure, after 30 days and after 12 months	7% procedure-related events after 30 days	2% stroke, < 1% systemic embolism	0% after 30 days	35% (for all atrial arrhythmias)	0.5%	N/A	N/A
	N/A	3.5% procedure-related events after 30 days	< 1% stroke, 0% systemic embolism	0% after 30 days	36% (for all atrial arrhythmias)	N/A	N/A	N/A
	N/A	P non-significant	Non-inferiority P < 0.001 for stroke and systemic embolism	N/A	*P* = 0.392	N/A	N/A	N/A
Romanov et al. (RCT) [38]	100%	0% peri-procedural complications	0%	0%	41%	0%	100% Warfarin	N/A
	N/A	2% peri-procedural complications	0%	0%	34%	N/A	100% Warfarin	N/A
	N/A	*p* = 0.38	N/A	N/A	*p* = 0.34	N/A	N/A	N/A
Sun et al. [39] A	100%	10.0% procedural	HFrEF vs HFmrEF: HR 0.189 for stroke/TIA/SE *p* = 0.414	HFrEF vs HFmrEF: HR 0.236 *p* = 0.505	HFrEF vs HFmrEF: HR 0.788 *p* = 0.653	6.7%	NOAC 15%, SAPT 70%, None 15% after 1 year	85%
	100%	8.8% procedural				6.5%	Warfarin 2.2%, NOAC 8.9%, DAPT 2.2%, SAPT 68.9%, None 17.8% after 1 year	88.9%
	*p* > 0.999	*p* > 0.999				*p* > 0.999	N/A	N/A
Sun et al. [39] B	100%	9.2% procedural	No procedure vs Combined procedure HR 3.774 for stroke/TIA/SE *p* = 0.032	No procedure vs Combined procedure HR 3.032 *p* = 0.129	N/A	N/A	N/A	N/A
	N/A	N/A			N/A	N/A	N/A	N/A
	N/A	N/A			N/A	N/A	N/A	N/A
Swaans et al. [13]	100% after the procedure, at 60 days and at 6 months	N/A	0%	0% periprocedural, 10% after 12 months	30%	0%	N/A	77%
Zhao et al. [14]	100% after the procedure and after 3 months	N/A	0%	0%	23.8%	0%	100% OAC	N/A

**Table 3:** Retrospective studies investigating combined catheter ablation and left atrial appendage occlusion

Author	Number of patients	Age	CHA_2_DS_2_-VASc	HAS-BLED	Ablation energy	LAAO Device	FU time	Procedural success
Beney et al. [40]	10	70 (62–76)	2.5 (2–4)	3 (1–4)	Pulsed Field	Watchman	9 (3–12) months	100%
Chen et al. 2022 [41]	1114 patients	70.1 ± 8.0	3.6 ± 1.5	2.4 ± 1.0	Radiofrequency	Watchman (993 patients), LACBES (121 patients)	1960 patient-years	N/A
Du et al. 2018 [6]	Occlusion-first group: 52 patients	66.1 ± 7.8	4.2 ± 1.3	3.5 ± 1.1	N/A	Watchman	N/A	100%
	Ablation-first group: 30 patients	66.3 ± 9.5	4.7 ± 1.4	3.4 ± 0.9	N/A	Watchman	N/A	100%
	Total: 82 patients	66.2 ± 8.4	4.4 ± 1.4	3.5 ± 1.0	N/A	Watchman	11.2 ± 7.3 months	100%
	*p*	*p* = 0.909	*p* = 0.108	*p* = 0.567	N/A	N/A	N/A	*p* = 1.000
Du et al. 2019 [42]	122 patients	66.4 ± 8.8	4.3 ± 1.4	3.3 ± 1.0	Radiofrequency	Watchman (83 patients), Amplatzer Cardiac Plug (39 patients)	11.5 ± 6.8 months	100%
Fei et al. 2023 [25]	Combined group: 252 patients	N/A	N/A	3.1 ± 1.2	N/A	Watchman	At least 1 year	100%
	LAAO-only group: 63 patients (52 after PSM)	N/A	N/A	N/A	N/A	Watchman	At least 1 year	100%
	CA-only group: 157 patients (141 after PSM)	N/A	N/A	1.6 ± 1.0	N/A	N/A	At least 1 year	100%
	*p*	N/A	N/A	*p* < 0.001	N/A	N/A	N/A	N/A
Fei et al. 2024 [29]	≥ 75 years old: 66 patients	78.2 ± 4.6	5.8 ± 1.3	3.1 ± 1.0	Radiofrequency	Watchman 66.7%, Amplatzer cardiac plug 18.2%, Lambre 15.2%	12.2 (6.7–24.4) for AF recurrence, 27.9 (9.3–44.8) for clinical outcomes	100%
	< 75 years old: 250 patients	65.4 ± 7.0	4.0 ± 1.4	2.8 ± 0.9	Radiofrequency	Watchman 69.6%, Amplatzer cardiac plug 15.2%, Lambre 15.2%	11.9 (5.5–23.6) for AF recurrence, 25.2 (10.8–45.7) for clinical outcomes	100%
	*p*	*p* < 0.001	*p* < 0.001	*p* = 0.094	N/A	*p* = 0.836	*p* = 0.365 for AF recurrence, *p* = 0.668 for clinical outcomes	*p* = 1.000
Hao et al. [28]	Combined group (after PSM): 83 patients	69 (67,72)	3.45 ± 0.16	2.18 ± 0.11	N/A	Watchman (65.1%), Amplatzer cardiac plug or Lambre or Lacbes (34.9%)	25 (20, 30) months	100%
	LAAO-only group (after PSM): 83 patients	69 (67,73)	3.37 ± 0.15	2.15 ± 0.10	N/A	Watchman (75.9%), Amplatzer cardiac plug or Lambre or Lacbes (24.1%)	24 (19, 30) months	100%
	*p*	*p* = 0.67	*p* = 0.74	*p* = 0.81	N/A	*p* = 0.11	*p* = 0.78	*p* > 0.99
Kita et al. [43]	First-time ablation: 20 patients	71.2 ± 9.4	3.3 ± 1.3	2.5 ± 1.5	Radiofrequency	Watchman	17.0 ± 9.9 months (for device-related results), 16.5 ± 22.0 months (for AF ablation results)	N/A
	Re-do ablation: 22 patients	71 ± 7.8	3.3 ± 1.0	2.5 ± 1.3	Radiofrequency	Watchman	20.1 ± 7.1 months (for device-related results), 19.0 ± 7.1 months (for AF ablation results)	N/A
	Total: 42 patients	71.1 ± 8.5	3.3 ± 1.1	2.5 ± 1.4	Radiofrequency	Watchman	18.6 ± 8.6 months (for both device-related and AF ablation results)	N/A
	*p*	*p* = 0.94	*p* = 0.96	*p* = 0.91	N/A	N/A	*p* = 0.24	N/A
Li et al. 2018 [23]	25 patients	64.2 ± 3.5	4.5 (2–6)	3.17 (1–7)	Radiofrequency	Watchman	Up to 1 year	100%
Li et al. 2019 [24]	AF recurrence group: 11 patients	67.8 ± 8.5	4.0 ± 1.4	1.8 ± 1.3	Radiofrequency	Watchman	6 months	100%
	Sinus Rhythm maintenance group: 42 patients	70.4 ± 7.5	3.6 ± 1.5	1.6 ± 1.0	Radiofrequency	Watchman	6 months	100%
	Total: 53 patients	69.9 ± 7.7	3.7 ± 1.5	1.6 ± 1.1	Radiofrequency	Watchman	6 months	100%
	*p*	*p* = 0.323	*p* = 0.345	*p* = 0.782	N/A	N/A	N/A	N/A
Li et al. 2020 [3]	Combined group: 61 patients	66.7 ± 9.2	4.2 ± 1.1	3.4 ± 1.1	Radiofrequency	Watchman	Up to 24 months	N/A
	LAAO-only group: 46 patients	67.1 ± 7.4	4.3 ± 1.1	3.6 ± 1.2	N/A	Watchman	Up to 24 months	N/A
	Total: 107 patients	66.9 ± 8.4	4.3 ± 1.1	3.5 ± 1.1	N/A	Watchman	Up to 24 months	N/A
	*p*	*p* = 0.739	*p* = 0.810	*p* = 0.241	N/A	N/A	N/A	N/A
Li et al. 2024 [27]	Combined group: 102 patients(after PSM)	70.6 ± 1.0	3.0 (2.0, 5.0)	2.0 (2.0, 3.0)	Cryoballoon	Watchman 88.3%, LAmbre 11.8%	37.7 ± 6.5 months	100%
	LAAO-only group: 51 patients (after PSM)	69.7 ± 1.1	4.0 (3.0, 5.0)	3.0 (2.0, 3.0)	N/A	Watchman 88.2%, LAmbre 11.8%	37.3 ± 10.4 months	100%
	Catheter ablation – only group: 57 patients	N/A	N/A	N/A	N/A	N/A	26.7 ± 7.9 months	N/A
	*p*	*p* = 0.532	*p* = 0.266	*p* = 0.083	N/A	*p* = 1.000 (for both devices)	*p* = 0.763	N/A
Ma et al. [26]	CBA & LAAO: 45 patients	61.4 ± 9.8	3 (1, 4)	1 (0, 2)	Cryoballoon	Watchman	12 months	100%
	RFA & LAAO: 67 patients	63.7 ± 8.4	3 (2, 4)	2 (1, 2)	Radiofrequency	Watchman	12 months	100%
	*p*	*p* = 0.199	*p* = 0.134	*p* = 0.036	N/A	N/A	N/A	100%
Meng et al. [20]	DSA-guided only: 71 patients	71.8 ± 7.8	3.0 (2.0, 4.0)	2.0 (1.0, 3.0)	Cryoballoon	Watchman	N/A	100%
	DSA and TOE guided: 67 patients	70.3 ± 8.7	3.0 (2.0, 4.0)	2.0 (1.0, 3.0)	Cryoballoon	Watchman	N/A	100%
	Total: 138 patients	71.0 ± 8.3	3.0 (2.0, 4.0)	2.0 (1.0, 3.0)	Cryoballoon	Watchman	24 months	100%
	*p*	*p* = 0.244	*p* = 0.586	*p* = 0.557	N/A	N/A	N/A	N/A
Mo et al. [17]	Combined group: 76 patients	69.9 ± 7.9	3.6 ± 1.3	3.3 ± 1.1	N/A	Watchman	23.3 ± 6.9 months for all groups	100%
	CA-only group: 76 patients	69.5 ± 7.8	3.4 ± 1.4	2.6 ± 0.9	N/A	N/A	23.3 ± 6.9 months for all groups	Ν/Α
	LAAO-only group: 76 patients	71.36 ± 8.9	3.7 ± 1.4	3.4 ± 1.1	N/A	Watchman	23.3 ± 6.9 months for all groups	100%
	P1 for combined and CA-only comparison P2 for combined and LAAO-only comparison	P1 = 0.703 P2 = 0.300	P1 = 0.484 P2 = 0.719	P1 < 0.001 P2 = 0.413	N/A	N/A	N/A	Ν/Α
Pasupula et al. [44]	Combined group: 375 patients	74 ± 9.2	3.8 ± 1.3	N/A	N/A	N/A	N/A	N/A
	LAAO-only group: 407 patients	74 ± 8.7	N/A	N/A	N/A	N/A	N/A	N/A
	CA-only group: 406 patients	74 ± 9.2	N/A	N/A	N/A	N/A	N/A	N/A
	*p*	*p*	N/A	N/A	N/A	N/A	N/A	N/A
Phillips et al. [22]	98 patients	65 ± 7	2.6 ± 1.0	1.9 ± 0.8	Radiofrequency	Watchman	802 ± 439 days (88 – 1932)	100%
Preisendörfer et al. [32]	Combined group: 72 patients	70.2 ± 7.3	4.2 ± 1.1	2.2 ± 0.8	Radiofrequency or Cryoballoon	Watchman 94.4%, Amulet 5.6%	70.8% 6-month follow-up	95.8%
	LAAO-only group: 144 patients	76.7 ± 6.9	4.4 ± 1.2	2.3 ± 0.7	N/A	Watchman 90.3%, Amulet 9.7%	90.3% 6-month follow-up	95.8%
	*p*	*p* < 0.001	*p* = 0.26	*p* = 0.34	N/A	*p* = 0.3 (for both devices)	*p* < 0.001	*p* = 0.99
Ren et al. 2020 [31]	Combined group: 42 patients	70 ± 7.6	3.8 ± 2.1	3.7 ± 1.2	Cryoballoon	Lefort 52.4%, Lacbes 26.2%, Watchman 21.4%	20 ± 9 months	97.6%
	Catheter ablation -only group: 262 patients	66.3 ± 9.5	2.8 ± 1.9	2.7 ± 1.2	Cryoballoon	N/A	22 ± 11 months	100%
	*p*	*p* = 0.01	*p* = 0.001	*p* < 0.0001	N/A	N/A	N/A	N/A
Ren et al. 2021 [30]	76	67.0 ± 7.5	3.4 ± 1.9	2.3 ± 1.1	Cryoballoon	Lefort 50.7%, Lacbes 22.7%, Watchman 26.7%	23.7 ± 11.0 months	97.4%
Yang et al. 2021 [18]	Combined group: 65 patients	61.8 ± 7.9	3 (2, 4)	3 (2, 3)	Radiofrequency	Watchman	12 months for all groups	100%
	CA- only group: 65 patients	60.7 ± 9.1	4 (3, 5)	3 (2, 3)	Radiofrequency	N/A	12 months for all groups	100%
	*p*	*p* = 0.47	*p* = 0.09	*p* = 0.95	N/A	N/A	N/A	N/A
Yang et al. 2022 [19]	Combined group: 62 patients	64.2 ± 8.3	3.8 ± 1.6	3 (1, 3)	Radiofrequency	Watchman	3 months for all groups	100%
	CA- only group: 62 patients	62.5 ± 7.2	3.3 ± 1.7	2 (1, 3)	Radiofrequency	N/A	3 months for all groups	100%
	*p*	*p* = 0.20	*p* = 0.10	*p* = 0.11	N/A	N/A	N/A	N/A
Zhang PP et al. 2023 [21]	< 80 years old: 459 patients	68.2 (6.9)	3.1 (1.4)	2.2 (1.1)	Radiofrequency	Watchman	27.1 ± 9.1 months	100%
	≥ 80 years old: 46 patients	82.3 (0.3)	4.1 (1.3)	2.2 (0.9)	Radiofrequency	Watchman	25.8 ± 8.7 months	100%
	Total: 505 patients	69.5 (7.7)	3.2 (1.4)	2.2 (1.1)	Radiofrequency	Watchman	26.9 ± 9.1 months	100%
	*p*	*p* < 0.001	*p* < 0.001	*p* = 0.98	N/A	N/A	*p* = 0.7	N/A
Zhang Y et al. 2023 [45]	Combined group: 283 patients	63.71 ± 7.75	3.87 ± 1.47	2.28 ± 1.25	N/A	63.3% Watchman, 33.2% LAmbre, 3.6% Amplatzer Cardiac Plug	N/A	N/A
	LAAO-only group: 78 patients	63.59 ± 9.21	3.94 ± 1.53	2.26 ± 1.21	N/A	60.3% Watchman, 38.5% LAmbre, 1.3% Amplatzer Cardiac Plug	N/A	N/A
	Total: 361 patients	63.63 ± 8.07	3.88 ± 1.48	2.27 ± 1.24	N/A	62.6% Watchman, 34.3% LAmbre, 3.0% Amplatzer Cardiac Plug	28.27 ± 15.00 months	N/A
	*p*	*p* = 0.91	*p* = 0.73	*p* = 0.90	N/A	N/A	N/A	N/A
Zhang X et al. 2023 [46]	Elderly patients (≥ 80 y.o.) on half-dose rivaroxaban: 83 patients	82.6 ± 1.7	6.4 ± 1.2	3.6 ± 0.9	Cryoballoon	Watchman 65%, Lacbes 35%	At least 1 year	100%
	Non-elderly patients (< 80 y.o.): 120 patients	69.2 ± 7.4	4.7 ± 1.7	3.07 ± 0.8	Cryoballoon	Watchman 54%, Lacbes 46%	At least 1 year	100%
	*p*	*p* < 0.001	p < 0.001	p < 0.001	N/A	*p* = 0.15	N/A	*p* = 1.000
Zhu et al. [4]	Combined group: 51 patients	64.4 ± 9.44	3.80 ± 0.95	3.50 ± 0.83	Radiofrequency	Watchman	25.2 ± 16.2 months	100%
	LAAO-only group: 47 patients	64.9 ± 8.62	3.65 ± 0.99	3.55 ± 0.89	Radiofrequency	Watchman	22.4 ± 18.8 months	100%
	Total: 98 patients	N/A	N/A	N/A	Radiofrequency	Watchman	N/A	100%
	*p*	*p* = 0.86	*p* = 0.63	*p* = 0.86	N/A	N/A	*p* = 0.15	*p* = 1

Author	Νο Residual leak or leak ≤ 5 mm	Total adverse events	Thromboembolic events by the end of FU time	Major bleeding events	AF recurrence	DRT	Antithrombotic treatment after the procedure	Freedom from OAC by the end of FU time
Beney et al. [40]	N/A	No safety endpoint occurred (only 1 minor hemoptysis)	0%	0%	30%	0%	100% OAC	100%
Chen et al. 2022 [41]	99.7% after 3 months	5.3% periprocedural	1.6% per 100 patient-years	0.9% per 100 patient-years	16.7% at 12 months, 26.6% at 24 months, 40.2% at 36 months, 43.2% at 48 months	1.9% at 3 months	65.3% antiplatelets, 13.3% OACs, 21.3% no therapy (at 4 years)	86.7%
Du et al. 2018 [6]	100% after the procedure and during follow-up	N/A	0% stroke or TIA	0%	25.0%	1.9%	N/A	100%
	100% after the procedure and during follow-up	N/A	0% stroke or TIA	0%	30.0%	3.3%	N/A	100%
	100% after the procedure and during follow-up	N/A	0% stroke or TIA	0%	26.8%	2.4%	N/A	100%
	N/A	N/A	N/A	N/A	*p* = 0.311	*p* = 0.690	N/A	N/A
Du et al. 2019 [42]	100%	N/A	0% stroke	0%	23.8%	1,6%	N/A	N/A
Fei et al. 2023 [25]	100%	N/A	0.4%	0% during hospitalization	21.8% after one year	0%	100% OAC before discharge	N/A
	100%	N/A	3.2%	0% during hospitalization	N/A	0%	100% OAC before discharge	N/A
	N/A	N/A	0.6%	0% during hospitalization	24,2% after one year	0%	100% OAC before discharge	N/A
	N/A	N/A	N/A	N/A	N/A	N/A	N/A	N/A
Fei et al. 2024 [29]	100%	N/A	1.5% stroke/TIA	1.5%	21.2%	1.5%	For both groups at discharge: 47 patients warfarin, 140 rivaroxaban, 129 dabigatran	N/A
	100%	N/A	1.6% stroke/TIA	3.2%	19.2%	1.2%		N/A
	*p* = 1.000	N/A	*p* = 0.609	*p* = 0.691	*p* = 0.729	*p* = 1.000		N/A
Hao et al. [28]	98.8%	4.8% periprocedural, 11,6% total events until end of follow-up	0%	2.4%	N/A	3.6%	N/A	N/A
	98.8%	2.4% periprocedural, 23.4% total events until end of follow-up	1.9%	0%	N/A	4.8%	N/A	N/A
	*p* > 0.99	*p* = 0.68 for periprocedural, *p* = 0.20 for total events until end of follow-up	*p* = 0.32	*p* = 0.16	N/A	*p* > 0.99	N/A	N/A
Kita et al. [43]	0% after the procedure, 5,2% after 3 and 6 months	N/A	0%	0%	30.0%	5.0% (at 3 or 6 months)	N/A	N/A
	0% after the procedure, 3 and 6 months	N/A	0%	0%	36.4%	9.1% (at 3 or 6 months)	N/A	N/A
	0% after the procedure, 2.4% after 3 months, 0% after 6 months	N/A	0%	0%	33.3%	7.3% (at 3 or 6 months)	N/A	N/A
	*p* = 0.48 at 3 and 6 months	N/A	N/A	N/A	*p* = 0.74	*p* = 1.0	N/A	N/A
Li et al. 2018 [23]	100%	8% minor periprocedural complications	4% TIA, 0% stroke	0%	56% at 6 months, 50% at 1 year	0%	100% OACs	96% at 6 months, 100% at 1 year
Li et al. 2019 [24]	100% after the procedure and at 3 months	N/A	0%	0%	100%	N/A	100% OAC before discharge	N/A
	100% after the procedure and at 3 months	N/A	0%	0%	0%	N/A	100% OAC before discharge	N/A
	100% after the procedure and at 3 months	N/A	0%	0%	20.8% between 3 and 6 months	N/A	100% OAC before discharge	N/A
	N/A	N/A	N/A	N/A	N/A	N/A	N/A	N/A
Li et al. 2020 [3]	100%	N/A	0% stroke, 4.9% TIA	6.6% total bleeding events	50.8% after 1 year	1.6% at 45 days	N/A	N/A
	100%	N/A	0%	2.2% total bleeding events	N/A	0%	N/A	N/A
	100%	N/A	0% stroke, 2.8% TIA	4.7%	N/A	0.9%	N/A	N/A
	N/A	N/A	N/A	N/A	N/A	N/A	N/A	N/A
Li et al. 2024 [27]	1% after the procedure, 0% at 12 months	5.9% periprocedural complications	4.0% overall thrombotic events, 3.0% ischemic stroke & TIA	1.0% periprocedural, 2.0% during follow-up	28%	0%	4.9% none, 7.8% SAPT, 17.7% Warfarin, 69.6% NOACs	91%
	0% after the procedure, 0% at 12 months	5.9% periprocedural complications	7.8% overall thrombotic events, 3.9% ischemic stroke & TIA	0% periprocedural, 2.0% during follow-up	N/A	0%	3.9% none, 9.8% SAPT, 21.6% Warfarin, 64.7% NOACs	92.2%
	N/A	N/A	N/A	N/A	15.8%	N/A	N/A	N/A
	N/A	*p* = 1.000	*p* = 0.301 for overall thrombotic events, *p* = 0.736 for ischemic stroke & TIA	N/A for periprocedural, *p* = 0.989 during follow-up	*p* = 0.075	N/A	N/A	N/A
Ma et al. [26]	100%	6.7% peri-procedural, 0.0% during follow-up	0%	0.0% peri-procedural, 0.0% during follow-up	40.0%	2.2%	100% OAC	88.9%
	100%	6.0% peri-procedural, 1.5% during follow-up	0%	4.5% peri-procedural, 1.5% during follow-up	40.3%	0%	100% OAC	77.6%
	100%	*p* = 1.000 (for peri-procedural as well as follow-up events)	*p* = 1.000	*p* = 0.272 (for peri-procedural events), *p* = 1.000 for follow-up events	*p* = 0.97	N/A	N/A	N/A
Meng et al. [20]	100% after the procedure and at 3 months	N/A	0%	1.5%	20.0%	0%	N/A	N/A
	100% after the procedure and at 3 months	N/A	4.8%	1.6%	28.6%	0%	N/A	N/A
	100% after the procedure and at 3 months	N/A	2.3%	1.6%	24.2%	0%	N/A	N/A
	N/A	N/A	*p* = 0.232	*p* = 1.000	*p* = 0.305	N/A	N/A	N/A
Mo et al. [17]	100% after the procedure	3.9% periprocedural and 3,9% during follow-up	1.3% ischemic stroke events	0% periprocedural and 2.6% during follow-up	32.9%	0%	96.0% OAC	97.4%
	N/A	2.6% periprocedural and 5,3% during follow-up	1.3% ischemic stroke events	0% periprocedural and 3.9% during follow-up	30.3%	N/A	100% OAC	38.2%
	100% after the procedure	2.6% periprocedural and 2,6% during follow-up	0% ischemic stroke events	0% periprocedural and 2.6% during follow-up	N/A	1.3%	97.4% OAC	N/A (similar to the combined group)
	N/A	P1 = 0.650 for periprocedural and 0.699 during follow-up P2 = 0.650 for periprocedural and 0.650 during follow-up	P1 = 1.000 P2 = 0.317	P1 = 0.650 during follow-up P2 = 1.000 during follow-up	P1 = 0.727	P2 = 0.317	N/A	N/A
Pasupula et al. [44]	N/A	8.1% (MACEs during hospitalization)	0% (during hospitalization)	3.1% (during hospitalization)	N/A	N/A	N/A	N/A
	N/A	5.3% (MACEs during hospitalization)	< 2.4% (during hospitalization)	< 2.4% (during hospitalization)	N/A	N/A	N/A	N/A
	N/A	7.4% (MACEs during hospitalization)	< 2.4% (during hospitalization)	< 2.4% (during hospitalization)	N/A	N/A	N/A	N/A
	N/A	*p* = 0.6086	*p* = 0.5819	*p* = 0.4552	N/A	N/A	N/A	N/A
Phillips et al. [22]	100% after the procedure, at 6 weeks and at 12 months	N/A	0.5% ischemic stroke rate	N/A	24% at 3 months, 23% at 12 months, 36% at 24 months	2% by the 6th week	100% OAC	100%
Preisendörfer et al. [32]	98.5% after 45 days	1.4% major hospital complications	0% cardioembolic events within 6 months	11.5% total bleeding events within 6 months	N/A	4.5%	93.1% SAPT & OAC, 6.9% OAC	88.2% after 6 months
	100% after 45 days	2.1% major hospital complications	0.8% cardioembolic events within 6 months	10.7% total bleeding events within 6 months	N/A	4.5%	9% DAPT, 88.2% SAPT & OAC, 2.8% OAC	96.2% after 6 months
	*p* = 0.33	*p* = 0.72 for major hospital complications	*p* = 0.55	*p* = 0.87	N/A	*p* = 0.96	*p* = 0.013	N/A
Ren et al. 2020 [31]	0% after the procedure, 0% after 3 and 12 months	N/A	0% (stroke)	0%	29.3%	0%	100%	N/A
	N/A	N/A	0.7% (stroke)	0.7%	15.3%	N/A	100%	N/A
	N/A	N/A	*p* = 1.00	p non-significant	*p* = 0.04 but non-significant after adjustment for confounding parameters	N/A	N/A	N/A
Ren et al. 2021 [30]	0% after the procedure, 1.4% at 12 months	N/A	1.4% stroke, 0% systemic embolism	1.4%	32.6% at 12 months, 48% at last follow-up	1.4%	21.1% Warfarin, 78.9% NOACs	97.2%
Yang et al. 2021 [18]	100% after the procedure and after 3 months	N/A	0%	1.54%	28%	1.54% after 3 months	100%	N/A
	N/A	N/A	0%	3.07%	25%	N/A	100%	N/A
	N/A	N/A	N/A	N/A	*p* = 0.69	N/A	N/A	N/A
Yang et al. 2022 [19]	100% after 3 months	N/A	0% stroke or TIA	0%	24.2%	2%	N/A	N/A
	N/A	N/A	0% stroke or TIA	2%	21.0%	N/A	N/A	N/A
	N/A	N/A	N/A	*p* = 0.32	*p* = 0.67	N/A	N/A	N/A
Zhang PP et al. 2023 [21]	100% after 3 months	N/A	1.7% (stroke or TIA), 78% risk reduction	0%	22.4%	0.9% at 3 months	35.3% Warfarin, 23.1% Dabigatran, 41.6% Rivaroxaban	N/A
	100% after 3 months	N/A	4.3% (stroke or TIA), 58% risk reduction	2.1%	21.7%	2.1% at 3 months	32.6% Warfarin, 23.9% Dabigatran, 43.5% Rivaroxaban	N/A
	100% after 3 months	N/A	1.2% (stroke or TIA)	0.2%	22.4%	1.0% at 3 months	35.0% Warfarin, 23.2% Dabigatran, 41.8% Rivaroxaban	N/A
	N/A	N/A	*p* = 0.23	*p* = 0.091	*p* = 0.99	*p* = 0.38	*p* = 0.94	N/A
Zhang Y et al. 2023 [45]	0% at 3 months	4.9% procedure-related complications	1.4% stroke or TIA	2.1% total bleeding events	28.3%	0.5%	97.2% Rivaroxaban, 1.4% Warfarin, 1.4% Rivaroxaban & Antiplatelet therapy	98.6%
	0% at 3 months	2.6% procedure-related complications	5.1% stroke or TIA	3.8% total bleeding events	N/A	5.1%	92.3% Rivaroxaban, 5.1% Warfarin, 2.6% Rivaroxaban & Antiplatelet therapy	96.2%
	0% at 3 months	4.4% procedure-related complications	2.2%	2.5%	N/A	1.7%	96.1% Rivaroxaban, 2.2% Warfarin, 1.7% Rivaroxaban & Antiplatelet therapy	98.1%
	N/A	P non-significant for any procedure-related complication	*p* = 0.048	*p* = 0.65	N/A	*p* = 0.01	N/A	*p* = 0.36
Zhang X et al. 2023 [46]	100%	1.2% periprocedural, 8.4% during follow-up	2.4% cerebral infarction / TIA	1.2%	30.1%	3.6%	83.2% oral rivaroxaban, 16,8% rivaroxaban & clopidogrel (after the procedure)(rivaroxaban 10 mg)	N/A
	100%	0.8% periprocedural, 9.2% during follow-up	1.7% cerebral infarction / TIA	4.2%	26.7%	1.7%	75% oral rivaroxaban, 25% rivaroxaban & clopidogrel (after the procedure)(rivaroxaban 20 mg)	N/A
	N/A	*p* = 1.000 for periprocedural, *p* = 0.53	*p* = 0.78	*p* = 0.22	0.59	0.38	N/A	N/A
Zhu et al. [4]	100% after the procedure, 2% after 6 weeks	N/A	0% stroke	0%	N/A	2% after 6 weeks	N/A	N/A
	100% after the procedure and after 6 weeks	N/A	0% stroke	0%	N/A	0% after 6 weeks	N/A	N/A
	100% after the procedure, 1% after 6 weeks	N/A	0%	0%	N/A	1% after 6 weeks	99% OAC	94% after 6 weeks
	*P* = 1 (after the procedure) *P* = 0.520 (after 6 weeks)	N/A	N/A	N/A	N/A	*p* = 1.0	N/A	N/A

**Table 4:** Meta-analyses investigating combined catheter ablation and left atrial appendage occlusion

Author	Number of studies and patients	Age	CHA_2_DS_2_-VASc	HAS-BLED	Ablation energy	LAAO Device	FU time	Procedural success
Han et al. [47]	13 studies, 952 patients	N/A	N/A	N/A	N/A	Watchman (937 patients), Amplatzer Cardiac Plug (15 patients)	22.83 months	99.37%
Hu et al. [48]	21 studies, 1708 patients	N/A	N/A	N/A	Radiofrequency (1299 patients), Cryoballoon (150 patients), Radiofrequency or Cryoballoon (21 patients), Unknown (238 patients)	Watchman (1273 patients), ACP (87 patients), Watchman or ACP (49 patients), Lefort (23 patients), Lacbes (11 patients), Watchman or ACP or Lambre (27 patients), Unknown (238 patients)	N/A	N/A
Jiang et al. [49]	18 studies, 1154 patients	N/A	N/A	N/A	Radiofrequency (1069 patients), Cryoballoon (35 patients), Radiofrequency or Cryoballoon (50 patients)	Watchman (864 patients), ACP or Watchman (284 patients), LAmbre (6 patients)	21 (1–38) months	98%
Junarta et al. [50]	8 studies, 1878 patients Combined group: 966 patients	N/A	N/A	N/A	Out of 6 studies mentioning the ablation energy: Radiofrequency (596 patients), Cryoballoon (42 patients)	Watchman (520 patients), LARIAT (404 patients), Lefort or Lacbes or Watchman (42 patients)	From 3 to 24 months	96% successful device occlusion for Watchman/Lefort/Lacbes, 84% for LARIAT
	CA-only group: 912 patients	N/A	N/A	N/A	Radiofrequency ( 417 patients), Cryoballoon (262 patients)	N/A		N/A
	*p*-value	N/A	N/A	N/A	N/A	N/A		N/A
Li et al. 2021 [51]	16 studies, 1428 patients	N/A	N/A	N/A	Radiofrequency (1118 patients), Cryoballoon (118 patients), Radiofrequency or Cryoballoon (192 patients)	Watchman (1300 patients), Watchman and ACP (128 patients)	Mean follow-up of all studies approximately 12 months +	100%
Wang et al. [52]	5 studies, 699 patients	N/A	N/A	N/A	Radiofrequency (121 patients), Radiofrequency or cryotherapy (77 patients), Cryoballoon (42 patients)	Watchman (121 patients), Watchman and ACP (21 patients), Watchman, LeFort and Lacbes (42 patients), Watchman, Lambre, Lagger and ACP (56 patients)	10.1 – 24 months	N/A
Zhang et al. 2022 [53]	COA group: 19 studies, 1504 patients	66.2 (64.8 – 67.6)	3.3 (2.9 – 3.6)	N/A	Radiofrequency (1281 patients), Cryoballoon (152 patients), Radiofrequency or Cryoballoon (71 patients)	Watchman (1068 patients), Watchman or ACP (277 patients), LAmbre (56 patients), Watchman or ACP or LAmbre (27 patients), Lefort or Lacbes or Watchman (76 patients)	At least 12 months	99.1%
	TCA group: 6 studies, 454 patients	64.7 (63.5 – 65.9)	2.3 (2.2 – 2.4)	N/A	Epicardial (137 patients), Hybrid (95 patients), Hybrid or Epicardial (222 patients)	AtriClip (454 patients)	At least 12 months	93.9%
	*P*-value	*P* = 0.209	*P* = 0.012	N/A	N/A	N/A	N/A	*P* = 0.001

Author	Νο Residual leak or leak ≤ 5 mm	Total adverse events	Thromboembolic events by the end of FU time	Major bleeding events	AF recurrence	DRT	Antithrombotic treatment after the procedure	Freedom from OAC by the end of FU time
Han et al. [47]	N/A	N/A	5.24%	6.95% (all events during follow-up, out of which only 16% were major)	32.89%	N/A	93.59% OAC	11.61%
Hu et al. [48]	N/A	N/A	N/A	N/A	N/A	1.2% (0.7% -1.8%)	N/A	N/A
Jiang et al. [49]	N/A	N/A	1% (stroke and TIA rate)	1% during follow-up	24%	0%	N/A	N/A
Junarta et al. [50]	100%	RR for major periprocedural complications of the combined procedure in relation to CA-only = 1.28 *P* = 0.75	RR for stroke or systemic embolism of the combined procedure in relation to CA-only = 0.78 *P* = 0.64	N/A	RR for arrhythmia recurrence of the combined procedure in relation to CA-onl*y* = 1.04 *P* = 0.73	0.5%	N/A	N/A
	N/A			N/A		N/A	N/A	N/A
	N/A			N/A		N/A	N/A	N/A
Li et al. 2021 [51]	100% after a mean follow-up time of 12 months +	N/A	1%	3% (procedure-related), 1% (long-term)	34%	N/A	N/A	N/A
Wang et al. [52]	N/A	Combined-procedure group: 10.8% perioperative complications	Combined-procedure group: 0.8%	Combined-procedure group: 1.4%	Combined-procedure group: 32%	N/A	N/A	N/A
		Control group (Catheter ablation alone): 9.4% perioperative complications	Control group (Catheter ablation alone): 1.5%	Control group (Catheter ablation alone): 2.1%	Control group (Catheter ablation alone): 22%			
		*P* = 0.05	*P* = 0.61	*P* = 0.65	*P* = 0.21			
Zhang et al. 2022 [53]	99.1% right after the procedure	N/A	0.4 per 100 patient-years (stroke rate)	3.0% (all post-procedural hemorrhage events)	N/A	N/A	N/A	N/A
	93.9% right after the procedure	N/A	0.1 per 100 patient-years (stroke rate)	1.6% (all post-procedural hemorrhage events)	N/A			
	*P* = 0.001	N/A	*P* = 0.504	*P* = 0.023	N/A			

Abbreviations: *ACP *Amplatzer Cardiac Plug, *AF *Atrial Fibrillation, *CA *Catheter Ablation, *CBA *Cryoballoon Ablation, *COA *Catheter Left Atrial Appendage Occlusion Combined with Ablation, *DRT *Device-related Thrombosis, *DSA *Digital Subtraction Angiography, *FU time *Follow-up time, *HFmrEF *Heart Failure with mildly reduced Ejection Fraction, *HFrEF *Heart Failure with reduced Ejection Fraction, *LAAO *Left Atrial Appendage Occlusion, *LMWH *Low Molecular Weight Heparin, *MACEs *Major Adverse Cardiovascular Events, *NOACs *Non-vitamin K Oral Anticoagulants, *OAC *Oral Anticoagulation, *PSM *Propensity Score Matching, *RFA *Radiofrequency Ablation, *SAPT *Single Antiplatelet Therapy, *SE *Systemic Embolism, *TCA *Thoracoscopic Surgical Left Atrial Appendage Clipping Combined with Ablation, *TIA *Transient Ischemic Attack, *TOE *Transoesophageal echocardiogram

Regarding the reverse sequence, when the device used for LAAO falls under the category of the “pacifier principle”, an additional disc is integrated to seal the ostium of the LAA from the left atrial side [2]. This impedes catheter maneuver with subsequent risk for insufficient pulmonary vein isolation (PVI), as well as linear ablation of mitral isthmus and LA anterior wall. The catheter used for ablation could also accidentally dislocate the LAAO device or even perform the ablation on its surface leading to perforation and leak [5]. Nevertheless, success rates of this procedure sequence have also been reported to reach 100% [6].

Du et al. [6] specifically studied the optimal sequence and reported similar complete device occlusions, AF-free success rates and major adverse events between the two possible combinations. However, significantly more PDLs were found in the CA-first group (26.7%) compared to LAAO-first (7.7%), with the procedural strategy being independently associated with new PDLs.

Finally, meta-analyses of studies investigating any procedural sequence have showed success rates as high as 98–100% [47, 49, 51, 53], defined as successful sealing of LAAO with no flow or a remaining jet less than 5 mm right after the procedure. Intriguingly, the acute success rate has been found significantly higher in the catheter combined procedure than its surgical counterpart (99.1% vs 93.9%, *p* = 0.001) [53].

### Ablation Energy and LAAO Device Selection

Even though cryoballoon ablation as a single procedure is noninferior and similarly safe to radiofrequency ablation [54], there are some concerns regarding its role in the combined procedure, as it is more likely to alter LAA ostium shape and therefore interfere with the subsequent LAAO [5]. Epidemiological data, however, have not yet proved this speculation. A meta-analysis by Wang et al. found no difference in the atrial arrhythmia recurrence rate, thromboembolic and bleeding events when different types of ablation energies were compared [52]. Similarly, Ma et al. [26] showed that radiofrequency and cryoballoon ablation combined with LAAO have similar AF recurrence rates, incidence of PDLs, as well as safety outcomes. The procedural time, on the contrary, was significantly shorter for cryoballoon ablation (*p* = 0.000).

Results regarding the efficacy of different LAAO device types have been few and conflicting. A meta-analysis of 1154 patients showed that the Watchman device had a slightly higher procedural success rate than the other devices combined, (99% and 96%, respectively) [49]. However, a retrospective single-center study compared the Watchman with the LAcbes device and found similar rates of periprocedural complications, device-related thrombus (DRT), complete peri-device seal, and complete endothelialization [41]. The Watchman device has also been compared with the Amplatzer Cardiac Plug, without significant difference being found regarding AF-free rate, complete LAA occlusion, new or persistent PDLs [42].

### Procedural Imaging

TOE or Cardiac Computed Tomography angiography (CCTA) should be performed before LAAO to rule out pre-existing thrombus and assess anatomic suitability, as well as 6–24 weeks after the procedure for DRT detection. Intraprocedural guidance should be performed with either TOE or intracardiac echocardiography (ICE) [2]. In most available studies of combined CA and LAAO, TOE was used. However, ICE [15], conventional angiography [55–57] and digital subtraction angiography (DSA) have also been studied as guiding methods during the combined procedures, with promising results.

## Combined Procedure and Peri-Device Leakage (PDL)

The complex anatomy of LAA makes its interventional occlusion susceptible to PDL, mainly provoked by the edema occurring during implantation, subsequent atrial remodeling, non-circular LAA orifice shape, post-implant device migration, off-axis deployment or deficient endothelialization [4, 7]. In isolated LAAO, residual PDL has been reported in 12.5%−34.3% of cases as detected by TOE, while the incidence rises at 31%−68.5% when CT is used [5]. Considering that LAA is susceptible to thrombus formation due to low blood flow, its inadequate closure could result to increased thromboembolic risk. Available data, however, is conflicting. In earlier, smaller studies, the presence of PDL was not correlated to thromboembolism [58, 59]. More recent studies, however, associated residual leakage to increased thromboembolism [60, 61], major bleeding [61], major adverse events [61], the composite endpoint of stroke, systemic embolism or cardiovascular death [62] and the composite endpoint of stroke, transient ischemic attack (TIA) or systemic embolism [63].

Several studies have dived into PDL occurrence in combined procedures. Data from the EWOLUTION and WASP registries showed a significant rise of PDLs from 2.9% at implant to 39% after 28 days, which is higher than the equivalent percentage generally observed after isolated LAAO [7]. Similarly, in another registry the complete sealing rate plummeted from 93% at implant to 70% at 3 months follow-up [9]. Finally, Liu et al. reports a slightly higher complete sealing rate, which was 91.8% at 6 months [36]. Further retrospective studies have found a new PDL incidence of 12.3–25.5% [4, 17, 42] at 45 days. The rates of satisfactory sealing and PDLs have been found similar between men and women [35], octogenarians and non-octogenarians [21], elderly ≥ 75 and < 75 years old [29], HFrEF and HFmrEF [39], as well as first-time and re-do CA [43]. In comparison to isolated LAAO, despite some investigators having found a similar rate of new PDLs [17], Li et al. [3] demonstrated a significantly higher degree of residual flow at 45 days in the combined procedure arm (45.9% vs 4.3%, *p* < 0.001). This difference persisted throughout the 12-month follow-up, despite a downward trend. Similarly, Zhu et al. [4] also showed that both the overall and the newly developed residual leaks were significantly higher after 6 weeks in the combined procedure (37.3% vs 17%, *p* = 0.04 and 25.5% vs 8.5%, *p* = 0.03, respectively). In the study of Hao et al. [28] the PDL incidence was once again significantly higher in the combined group rather than isolated LAAO (43.4% vs 25.3%, *p* = 0.01), although PDLs > 5 mm were similar between the groups (1.2% vs 1.2%, *p* > 0.99). On the contrary, another retrospective analysis [32] found a trend towards more PDLs in the LAAO-only patients as compared to the one-stop procedure patients 45 days after the procedure (18.2% in combined arm vs 30.4% in controls, *p* = 0.07). Additional data derived from randomized trials are so as to better understand the effect of the combined procedure in the PDL incidence.

Phillips et al. [22], in a retrospective analysis of 98 combined procedures, detected 28 new PDLs at the 6-week TOE, out of which 10 persisted after 12-months. Interestingly, these events were associated with device angulation and “shoulder” protrusion, highlighting the importance of optimal device seating. Additionally, PDL persistence was correlated with a significantly lower amount of device compression (12 ± 3), as opposed to complete occlusion (15 ± 5). The results of this study suggest that, when feasible, oversizing of the device may be preferred, especially for patients whose device release site is closer to the LA ridge [4]. Currently, there are no guidelines regarding this matter, however, the EHRA/EAPCI expert consensus statement on catheter-based LAAO proposes that approximately 20–25% oversizing may reduce the likelihood of PDLs [2]. Regarding the combined procedure, Phillips et al. recommends oversizing by at least 15% [22]. Despite these recommendations, device sizing remains a topic of debate, awaiting further studies, as oversizing could increase the possibility of incomplete device expansion, and thus of device embolization [4, 64].

Regarding the use of CT, a study including 84 patients showed that 60.7% of cases did not develop PDL at 6 months, whereas 36.9% presented with a leak < 3 mm and 2.4% with a leak of 3–4.9%. Despite failing to demonstrate any correlation between PDL and short-term adverse events, it gave prominence to the role of CT in measuring LAA orifice, whose maximum diameter independently predicts the occurrence of postoperative PDL [14].

Aiming to summarize some of the available data, a meta-analysis of 952 patients demonstrated a 9.1% rate of residual flow in the periprocedural period, while the equivalent rate during TOE follow-up after a mean of 5.2 months was 15.4%. Patients with higher residual flow had significantly more procedural bleeding events, as well as a tendency towards increased all-cause mortality [47].

Finally, it has been postulated that adding concomitant LAA electrical isolation on top of the combined procedure, could facilitate closure of small PDLs due to decreased LAA contraction [43]. However, this theory has not been tested yet.

## Overall Adverse Events

Both interventions have been associated with some major complications, which have been significantly reduced over the last decade. In isolated CA, overall procedure-related complications are estimated to occur in 4.51% of the patients, 2.44% out of which are severe, 1.31% are vascular, 0.78% are pericardial tamponade/effusion and 0.17% are stroke/TIA [65]. On the other hand, the perioperative complications of isolated LAAO reached 8.4% in PROTECT-AF [66], but the rate has now dropped due to procedure optimization to as low as 2.7% [34] and 1.44% [67]. A meta-analysis on PROTECT-AF, PREVAIL and PRAGUE-17 trials demonstrated a 6.1% rate of stroke/systemic embolism, 10.5% major bleeding, 5.4% cardiovascular mortality and 14.3% all-cause mortality after a mean follow-up of 38.7 ± 17.2 months [68].

Regarding combined procedures, literature has demonstrated remarkable peri-procedural and follow-up safety, and potentially even more favorable, than isolated procedures, outcomes [7, 45]. The clinical trial aMAZE [37] randomized patients in a 2:1 ratio to undergo either the one-stop procedure or catheter ablation alone and found that the rates of procedure-related events after 30 days were similar between the groups (7% and 3.5% correspondingly, *p* > 0.05). Data from two registries showed an 8.7% rate of serious adverse events in the first 30 days, out of which only 1.4% was device or procedure-related [7]. A more extended follow-up revealed a mortality rate of 0.36 per 100 patient-years [8]. The registry of Wintgens et al. demonstrated a complication-free rate as high as 92.8% during the first 30 days, with serious adverse event rate being estimated to 2.2% and no reported deaths [9]. Similar low complication rates have been confirmed in other registries as well [35, 36].

The incidence of periprocedural complications in prospective observational studies ranges between 3%–9.2% [10, 11, 15, 16, 39], while adverse events have been estimated to occur in 5.7% of cases during a mean follow-up time of 13 months [16]. Similarly, retrospective studies have found an incidence of periprocedural complications ranging between 3.9%–8.1% [17, 26, 27, 41, 44, 45], with adverse events reported at 1.5–3.9% of patients [17, 26]. Furthermore, when comparing the combined procedure with the isolated CA and LAAO procedures, no statistically significant differences were observed [17, 27, 28, 32, 44, 45]. More specifically, Mo et al. [17] reports 3.9% periprocedural adverse events and 3,9% follow-up events in the combined procedure group, whereas the corresponding incidence rates were 2.6% and 5,3% for the CA-only group and 2.6% and 2,6% for the LAAO-only group. All *P*-values were non-significant. Similarly, Li et al. [27] found that periprocedural adverse events were observed in 5.9% of patients both in the one-stop procedure and in the LAAO-only group (*p* = 1.000). Regarding major hospital complications, Preisendörfer et al. [32] report an incidence of 1.4% in the combined procedure arm and 2.1% in isolated LAAO (*p* = 0.72).

Regarding gender differences, no significant difference in total and major periprocedural complications was found between sexes, but women were more likely to present with minor complications. Follow-up events were also similar between men and women. Both sexes have demonstrated significant improvement in quality of life (QoL), the extent of which was greater in women. Thus, the decision on whether to perform the combined procedure should be irrelevant to sex [35]. No difference in adverse events has also been found when comparing octogenarians with non-octogenarians [21], elderly ≥ 75 with < 75 years old [29] and HFrEF with HFmrEF patients [39], but the evidence is yet weak.

Regarding meta-analyzed data, Jiang et al. reports a 0% all-cause mortality and 0% cardiac/neurological mortality rate in 1154 patients [49]. In reference to periprocedural adverse events, Li et al. found a 0% rate of phrenic nerve palsy, intracoronary air embolism, device embolization, pericardial effusion and death, as well as a 3% rate of procedure-related bleeding events. The long-term adverse events were 0% for device dislocation, intracranial bleeding, pericardial effusion and all-cause mortality, as well as 1% for device embolization and bleeding events [51]. Han et al. in their meta-analysis report a 3.15% rate of pericardial effusion and a 5.02% rate of total bleeding events during the periprocedural period, while the long-term rates were 2.15% for all-cause mortality, 5.24% for thromboembolic events and 6.95% for bleeding events [47]. Junarta et al. [50] found no difference in major periprocedural complications between the combined procedure and CA alone (RR 1.28, *p* = 0.75). Similarly, another meta-analysis found no significant difference in perioperative complications between the combined and the catheter ablation alone procedures, however the result was borderline (*p* = 0.05) and in favor of the catheter ablation alone [52].

Finally, using the same septal puncture could theoretically increase the risk of iatrogenic atrial septal defect due to increased manipulations, however current evidence has failed to demonstrate this possibility [45]. Based on the above, the addition of LAAO to CA for AF does not increase the rate of major adverse events in the hands of experienced operators, with LAAO addition potentially benefiting specific high-risk patients.

## Clinical Efficacy

### Effect on Left Atrial Mechanical and Endocrine Function

When LAAO is performed individually, its effect on LA function is controversial. A meta-analysis of the available studies showed a significant increase in LA emptying fraction, but no difference in LA volume, peak atrial longitudinal strain, peak atrial contraction strain, strain during atrial contraction, as well as strain during ventricular systole and diastole [69]. However, some other studies have demonstrated a significant increase in left atrial volume (LAV) [70, 71], which has been connected to major adverse cardiovascular events (MACEs) [72–74]. Successful CA, on the other hand, is known to exert the opposite effect and significantly decrease LAV [75, 76].

In the retrospective analysis of Yang et al. [18], both combined procedure and isolated CA groups showed significant decreases in LA diameter and volume. Moreover, the mean LA total emptying fraction, LA expansion index and atrial emptying fraction significantly increased during the 12-month follow-up, with the improvements being similar between the groups. LA function was also assessed by strain indices, with the combined procedure showing a significant improvement in LA reservoir, conduit and contractile function after 1 year. These indices were also improved in the isolated ablation group, except for conduit function. LA reservoir tended to improve only in the isolated ablation group after 3 months, however at 1-year follow-up no significant difference was found. Since LA function improved in both groups, most of these beneficial effects potentially result from ablation rather than LAAO. Nevertheless, addition of LAAO did not interfere with LA function improvement, as it only resulted in an early, transient decline in LA function, which is justifiable considering LAA role in cardiac hemodynamics.

Another retrospective study [19] showed that LA ejection fraction (LAEF), longitudinal LA strain and LA strain rate increased continuously over a 3-month follow-up in both combined procedure and isolated CA. However, isolated CA was correlated with significantly higher values of longitudinal LA strain and strain rate, which reflects LA reservoir function. According to these results and in contrast to Yang et al. [18], adding LAAO to CA as a one-stop procedure possibly affects the ability of LA to store pulmonary venous return during left ventricular contraction and isovolumetric relaxation.

Furthermore, Li et al. [24] has reported the occurrence of LA structural reverse remodeling, defined as a reduction in LAV, in patients with maintained SR after the combined procedure. They found a significantly decreased LAV in this patient group 6 months after the intervention (130.2 ± 36.3 mL to 107.1 ± 30.0 mL, *p* < 0.001). Incidence of LA structural reverse remodeling was 100% in the SR maintenance group, but only 36.4% in those with AF recurrence (*p* < 0.001). Smaller preoperative LA Appendage Volume / LAV ratio (LAAV/LAV ratio), higher NT-proBNP and lower left ventricular ejection fraction (LVEF) were all significantly associated with LA structural reverse remodeling.

Interestingly, the retrospective study of Fei et al. [25] also investigated the effect of the combined procedure on LA reverse remodeling by including LA sphericity (LASP). Despite conflicting results [77], LASP has been independently associated with AF recurrence and thromboembolic events, as a more symmetric structure facilitates slow flow and stasis [78]. In the present study, LAV significantly decreased in the combined and CA-only groups, but it significantly increased in the LAAO-only group. Regarding LASP, it significantly increased in the combined and in the CA-only groups, but not in the LAAO-only group. Postprocedural LASP changes (Δ-sphericity), however, were similar between the combined and the LAAO-only groups, meaning that the addition of LAAO to CA could alleviate spheroidization from the ablation scar. Importantly, patients with neither volumetric nor spherical reverse remodeling had a significantly higher MACE rate. The beneficial effect of LA structural reverse remodeling on AF recurrence rates [79] and prognosis [80, 81] has already been found after CA and it remains to be investigated if a similar conclusion can also be drawn for the combined procedure.

Finally, it has been demonstrated that LVEF significantly increases after the combined procedure in patients without AF recurrence, contrary to patients with AF recurrence or isolated LAAO [27]. As the effect of the individually performed LAAO on the LVEF is controversial, with studies demonstrating opposing results [82, 83], this study suggests that the maintenance of SR in the combined procedure is potentially beneficial to the cardiac function.

In respect to cardiac endocrine function, LAA normally secretes B-type natriuretic peptide (BNP) [84]. As LAAO leads to LAA flow limitations, it could alter BNP secretion. A retrospective study [19], though, found that BNP levels were similar among patients undergoing the combined procedure and isolated CA. More specifically, BNP levels rapidly decreased after SR restoration of SR, rose until the third postoperative day and had a downward trend for the next 3 months in both arms. Therefore, concomitant to ablation LAAO does not seem to impede cardiac endocrine function.

### Stroke Incidence Reduction

LAAO significantly reduces stroke risk compared to the expected CHA_2_DS_2_-VASc score risk and is non-inferior to warfarin use [85]. However, data on CA are insufficient and rather controversial, with the CABANA trial [86] reporting that AF ablation, compared with medical therapy, does not significantly reduce disabling stroke rate. Nevertheless, the combination of CA and LAAO could significantly lessen stroke events.

The aMAZE trial showed that the combined procedure was non-inferior to CA-only in terms of any-cause stroke, but stroke prevention was not a study end point and future trials for this outcome are warranted [37]. The registry of Phillips et al. reports a stroke rate of 0.36 per 100 patient-years, which represents a 93% relative risk reduction as predicted by the CHA_2_DS_2_-VASc score for patients not taking OAC. When TIA and systemic embolism are added to stroke as a combined endpoint, the rates are 1.09 per 100 patient-years, translating to a 84% relative risk reduction [8]. Another registry reports a 0.7% annualized stroke rate (78% risk reduction) and 1.3% annualized composite thromboembolism rate (72% reduction) during a median follow-up of 34.5 months. Intriguingly, the risk reduction was observed despite many patients having relapsed into AF and 85% taking only antiplatelet or no antithrombotic therapy [9].

Data from prospective studies regarding the combined procedure have demonstrated a 1.2–4.8% [10, 12] stroke rate, a 2.9% TIA rate [16], while others found no thromboembolic events during a follow-up time of at least 6 months [11, 13–15]. Sun et al. [39] prospectively compared no-procedure with combined-procedure patients in regard to stroke/TIA/systemic embolism incidence and calculated a HR of 3.774 (*p* = 0.032), with the one-stop procedure therefore displaying a protective effect.

Retrospective studies have found similar rates of thromboembolic events ranging between 0 and 4% [6, 18, 20, 25–28, 32, 41, 43, 44], as well as strokes (0%–1.4%) [3, 4, 17, 22–24, 30, 31, 42], TIAs (0%–4.9%) [3, 19, 23], and strokes/TIAs (0%–3%) [19, 21, 27, 45], with no significant difference compared to isolated LAAO [4, 17, 27, 28, 32, 44] or CA [17–19, 44]. Mo et al. [17] reports 1.3% ischemic stroke events for the combined procedure group, 1.3% for the CA-only group and 0% for the LAAO-only group. Pasupula et al. [44] found a 0% rate of stroke/TIA/thromboembolism in the combined procedure cohort and < 2,4% in CA and LAAO-only cohorts, while Hao et al. [28] 0% in the combined procedure and 1.9% in LAAO-only (all *p*-values non-significant). An exception is the retrospective analysis of Zhang et al. [45], in which the combined procedure was significantly correlated with less stroke/TIA events than isolated LAAO (1.4% vs 5.1%, *p* = 0.048). This observation was assumed to be caused by the SR restoration in the first group, which in turn led to better LA blood flow direction and less platelet aggregation.

Finally, meta-analyzed data have demonstrated a 1% stroke/TIA rate [49], a 1% stroke/TIA/systemic embolism rate with a 79.7% average annualized stroke rate reduction [51] and a stroke incidence of 0.4 per 100 patient-years [53]. Intriguingly, the latter study also demonstrated a comparable stroke rate between the transcatheter combined procedure and surgery [53]. Accounting for antithrombotic treatment, Wang et al. also found similar thromboembolic events between the combined and isolated CA groups [52]. Another meta-analysis of 1878 patients also showed no significant difference in stroke or systemic embolism between the combined procedure and catheter ablation alone (RR 0.78, *P* = 0.64) [50].

### Arrhythmia Incidence Reduction

Success rates of CA in AF range between 30–75% at one year, with many patients requiring reintervention and developing late recurrences [1]. In a randomized trial by Romanov et al. [38] the combined procedure resulted in higher AF burden in the first 3 months, when compared to CA alone. Contrary, freedom from atrial tachyarrhythmias was similar after 3 months until last follow-up (59% versus 66%; *p* = 0.34). The authors attributed the early AF burden increase to LAA inflammation, mechanical irritation and lack of its electrical isolation. Another clinical trial [37] demonstrated that primary effectiveness, defined as absence of any atrial arrhythmias 12 months after the intervention, was 64.3% with the combined procedure and 59.9% with CA-only. The difference, however, did not meet the statistical criterion to establish superiority.

Registries report a 51% AF recurrence rate after 3 years [9] and 36% after 22 months, with the recurrence being significantly higher in persistent than paroxysmal AF [36]. No significant difference between ablation energy was found [36]. AF recurrence rates range between 22–42% in prospective studies [10–16] and 21.8%–50.8% in retrospective studies [3, 6, 17–23, 25–27, 30, 31, 41–43, 45], with no difference between the combined procedure and CA alone [17–19, 27]. More specifically, Mo et al. [17] have found a 32.9% AF recurrence rate in the combined procedure group and a 30.3% rate in the CA-only group (*p* = 0.727). In a similar way, Yang et al. [18] demonstrated a 28% AF recurrence rate in the combined procedure group and a 25% rate in the CA-only group (*p* = 0.69). An exception is the retrospective study of Ding et al. [87], in which the AF or atrial tachycardia recurrence was recorded significantly earlier in the combined group than in CA-only (*p* = 0.026). The researchers attributed this difference to the higher rates of insufficient ablation observed in the one-stop procedure, which was correlated with a reduction in the number of additional ablations, ablating attempts and ablation duration. When the insufficient ablation was corrected, the AF recurrence reduced.

Finally, a meta-analysis of 1154 patients demonstrated a 24% AF recurrence rate during an average follow-up of 21 months, with it being higher with the Watchman device and in the extended follow-up subgroup [49]. Similarly, other meta-analyses have found a 32.9% AF recurrence rate [47], 34% atrial arrhythmia recurrence rate [51], with no significant difference between the combined procedure and isolated CA (*p* = 0.21) [52]. Similar arrhythmia recurrence rates have also been reported by Junarta et al. [50], with a risk ratio of 1.04 (*p* = 0.73).

Despite no significant sex differences have been found in the recurrence rate of atrial tachyarrhythmias, a trend has been demonstrated towards greater AF recurrence rate in women, as in isolated CA studies [35], that deems further investigation.

### Bleeding Incidence Reduction and Suitable Antithrombotic Regimens

After LAAO, device endothelization initiates and decreases thrombus formation. However, this process can take up several weeks, creating a high-risk time frame for DRT. The incidence of DRT has been found to range from 3.7%−5.5% with the Watchman and 3.2–16.7% with the Amplatzer device [88], while it has been reported to be 1.2% in the combined procedure [48]. Interestingly, a retrospective study found the DRT incidence is significantly lower in the combined procedure, compared with isolated LAAO [45]. Nevertheless, antithrombotic treatment in the initial phase after LAAO is mandatory to prevent DRT, albeit its duration and type are not uniform across the different studies and registries. CA, on the other hand, has a better-established strategy, with the European Society of Cardiology recommending systemic anticoagulation with warfarin or NOAC for at least 2 months after the procedure and possible extended use based on the patient’s stroke risk profile [1].

When the one-stop procedure is performed, SR restoration could improve left ventricular function and subsequently decrease hemostasis and thrombus formation, however, acute tissue injury and prolonged procedure time could increase thrombogenesis [5]. Most patients undergoing the combined procedure receive the same antithrombotic strategy as patients undergoing the LAAO procedure alone, with a slight increase in the OAC duration (45–90 days).

In the aMAZE clinical trial no major bleeding events were observed neither in the combined procedure nor in the CA-only group 30 days after the intervention [37]. In a cohort study of two registries, OACs were used post-procedurally in 92.8% of patients, with 2.9% of serious bleedings at 30 days, which were equally distributed between NOACs and warfarin, while the rate was similar to isolated CA [7]. The rate of DRT was 2.1% at early follow-up TOE. After 2-years follow-up, only 8.0% of patients were still receiving OAC, while 81.9% were on antiplatelet therapy and 10.1% were not receiving any antithrombotic therapy. With a mean follow-up time of 726 ± 91 days, the observed major bleeding event rate was 1.83 per 100 patient-years (relative risk reduction of 70% for non-procedural bleedings predicted by the HAS-BLED score). This significant reduction is most probably explained by OAC discontinuation [8]. Similarly, another registry demonstrated a 3.2% major bleeding event rate at 34.5 months, which translates into a 1.1% annualized rate (HAS-BLED expected bleeding rate 3.7%). Only 15.1% of patients remained on OAC at last follow-up [9].

Prospective observational data agree with the previous studies, as major bleeding events rates hover between 1.4%−4.8% [10, 12, 15, 16]. Only one study exceeded these rates (10%) [13]. Sun et al. [39] prospectively compared no-procedure with combined-procedure patients regarding major bleeding event incidence and found a HR of 3.032 (*p* = 0.129). The percentage of patients freed from OACs ranged between 77–97% [10, 11, 13, 15, 16]. In retrospective studies, major bleeding events ranged between 0–4.5% [17, 25–27, 44] and 0–2.6% for periprocedural and last follow-up, respectively [4, 6, 17–21, 23, 24, 26, 27, 30, 31, 41–43]. The percentage of patients freed from OACs by the end of follow-up was 77.6% −100% [6, 17, 22, 23, 26, 27, 30, 41, 45]. Importantly, no significant difference has been found in the bleeding events between the combined and isolated procedures [17, 19, 27, 28, 31, 32, 44, 45]. Zhang et al. [45], after 12 months of follow-up, report 2.1% total bleeding events in the one-stop procedure and 3.8% in the LAAO-only (*p* = 0.65), while Preisendörfer et al. [32] 0% and 0.8% respectively within a follow-up time of 6 months (*p* = 0.87). Mo et al. [17] found no periprocedural bleeding events in any group (combined procedure, CA-only, LAAO-only), while in the follow-up time, they observed a 2.6% bleeding event rate in the combined and LAAO-only group, as well as a 3.9% rate in the CA-only group (p non-significant for all comparisons). Regarding elderly patients ≥ 80 years of age and anticoagulation after the one-stop procedure, it has been shown that lowering rivaroxaban to half-dose is a safe option [46].

Finally, meta-analyses have demonstrated a rate of major hemorrhagic complications equal to 1% [49, 51] and a 60.3% average annualized bleeding rate reduction based on the HAS-BLED score [51]. Han et al. further demonstrated a 6.9% rate of total bleeding events, out of which only 16% were major, while OAC use decreased from 93.6% to 11.6% after the combined procedure [47]. Notably, the meta-analysis of Wang et al. found similar bleeding events between combined and isolated CA (RR = 0.67, *p* = 0.65) [52]. Considering these results, an ongoing randomized controlled trial aims to determine whether LAAO with the Watchman device is a reasonable alternative to OAC after CA in AF patients (NCT03795298) [89]. The results are expected to better establish the role of the combined procedure in a more extended spectrum of clinical settings.

### Cost Effectiveness

Unsurprisingly, the combined procedure’s overall cost is less than the two independently staged procedures, mainly due to single hospital stay and operating room use. Moreover, Kawakami et al. [90] has shown the cost-effectiveness’ superiority of the combined procedure compared to long-term OAC by using a Markov model of hypothetical patients with symptomatic AF and high thrombotic and bleeding risk. In the base-case cohort of 10.000 patients followed for 10 years, total costs were 29.027$ for the LAAO procedure and 27.896$ for OAC strategy, with the former being associated with 122 fewer disabling strokes and 203 fewer intracranial hemorrhages. The cost-effectiveness ratio of CA + LAAO was estimated at 11.072 / quality-adjusted life-years (QALYs), with the cost-effectiveness rising in patients with higher stroke risk.

### Combined Procedure with Pulsed Field Ablation (PFA)

Most studies, to date, have evaluated cryoballoon or radiofrequency ablation in combination with LAAO. However, recently, another method for performing AF ablation has been introduced, namely pulsed field ablation (PFA). PFA has been developed as a non-thermal irreversible electroporation method to induce cardiac cell death and therefore ablation of the desired cardiac area, whilst reducing thermal injuries during the procedure [91, 92]. In terms of safety and efficacy, a recent meta-analysis including 18 studies and approximately 5000 patients concluded that PFA, compared to thermal ablation, has better short- and long-term efficacy as well as lower rated or thermal injuries, with, however, a potential increased rate of tamponade during the procedure [93]. Along with a similar safety and efficacy profile, PFA is a promising alternative for combined interventions as it requires significantly less procedural time than cryobaloon or RF ablation, while also induced less tissue edema, which could resolve any issues related to LAAO device sizing [94]. The first experience with the concept of combined PFA + LAAO procedure was introduced in a case report by Tang et al. [95], using a novel device that combined PFA and mechanical LAAO (E-SeaLA, Hangzhou Dinova EP Technology Co, Ltd). The authors reported no procedural complication, while the patient remained on sinus rhythm during the in-hospital course. Followingly, Beney et al. [40], reported their center’s experience with a combined PFA + LAAO, compared to isolated PFA and isolated LAAO, with the use of dedicated PFA and LAAO devices. 10 patients were enrolled in the combined intervention arm, being compared with 207 patients in the isolated AF and 61 patients in the isolated LAAO cohorts. No death, stroke, major bleeding event or pericardial effusion occurred. Regarding procedural times, total median procedure duration was 79 min (range 60–125) for the combined procedure, 71 min (25–241) for isolated PFA and 47 min (15–162) for isolated LAAO, while total fluoroscopy times were 21 min for the PFA + LAAO procedure (15–26), 15 min for the isolated PFA (5–44), and 10 min (3–50) for the isolated LAAO. Therefore, this study documented the safety of concomitant PFA + LAAO, which has comparable duration with isolated procedures. Given the promising results, less complications, increased efficacy of PFA in AF management, future studies evaluating further clinical and AF endpoints are needed, in order to establish the role of this technique in combined interventions.

## Indications and Future Directions

There are currently no specific guidelines for the combined CA and LAAO procedure. The updated 2020 EHRA/EAPCI expert consensus statement on catheter-based LAAO emphasizes the need for both procedures to be individually indicated to be concomitantly performed, with the combined procedure thus not being separately indicated per se [2]. There are, nonetheless, a handful of patients which could benefit from both the procedures. These are the patients who meet at least one of the recommendations for the performance of each procedure separately, including symptomatic non-valvular AF patients (1) whose antiarrhythmic drug or beta-blocker treatment has failed / is not tolerated or (2) whose first-line treatment is chosen to be CA when AF recurrence is of low probability or (3) in AF patients with HFrEF or tachycardia-induced cardiomyopathy, who have a CHA_2_DS_2_-VASc score ≥ 2 in males or ≥ 3 in females and (i) have a contraindication for OAC or (ii) an elevated bleeding risk under chronic OAC or (iii) are non-compliant to OAC therapy or (iv) OAC is not efficient.

Performing the CA and the LAAO simultaneously rather than sequentially is a considerable choice as it entails significant benefits (Graphical Abstract). It is cost-effective and requires only one vascular access, one transseptal puncture, once-performed anesthesia and theoretically less consequent risk of procedure-related complications, fewer total hospitalization days, shorter total required duration of OAC, as well as improved compliance and quality of life for the patients. Even though the available data comparing the combined procedure to the staged ones is still limited, there is strong evidence that their combination does not undermine the clinical results expected by the individual performance of each of them in terms of stroke, arrhythmia and bleeding incidence reduction. Nevertheless, most of the existing studies on the one-stop procedure are observational and lack randomization to a control group, either to LAAO or CA or OAC therapy alone. The lack of controls limits the power of the outcomes and does not allow to draw definitive and generalizable conclusions regarding efficacy and safety. Moreover, there is increased risk of selection bias and influence by confounding variables. Patients chosen for the combined procedure might have been relatively healthy compared to patients undergoing only CA or LAAO. On the other hand, the LAAO procedure could have been routinely recommended for patients in need of oral anticoagulation but unable to receive it due to contraindications, including comorbidities related to increased bleeding risk. Regarding catheter ablation, the history of a previous ablation procedure could also potentially affect the results of the studies. Even though the majority of the retrospective studies performed propensity score matching, there are still cases of unmatched covariates such as comorbidities, social demographics and medication non-compliance. Last but not least, the majority of the available cohort studies have included a limited number of patients with a relatively short follow-up, making it difficult to evaluate long-term or rare outcomes and complications. To organize randomized studies is understandably difficult as there are specific indications for each type of treatment and submitting a patient to an additional procedure or omitting an indicated one is ethically questionable. Randomization, however, to either the combined or the two-staged procedures could be a rational solution. Therefore, future randomized studies need to address the aforementioned issues, while being more powered, in order to optimally evaluate clinical outcomes. Understanding logistic and ethical limitations, future randomized studies should aim to initially include patients undergoing the combined procedure with at least one intervention indication, while the control group should receive the optimal medical therapy. As omitting to provide both interventional treatments in patients with both indications (i.e. indication for ablation and unsuitable for anticoagulation) is not easily performed, large registry and single-arm studies could also provide some further insight. Moreover, further studies with emerging technologies and designs, such as the concomitant LAAO and ablation device previously described with PFA or using transcatheter left atrial appendage exclusion [96], rather than occlusion devices, are necessary in order to potentially improve outcomes. In this context, there are currently two ongoing clinical trials testing the safety and efficacy of combined ablation and LAAO with the use of a single PFA + LAAO device (NCT05731882) or with an isolated PFA catheter and a Watchman occluder (NCT05560204), while another study, the LAACablation (NCT03788941), aims to investigate the safety and efficacy of the combined procedure, while reporting its impact on quality of life. Finally, the OPTION trial (NCT03795298), comparing LAAO with anticoagulation after ablation, includes a randomized cohort in a subset of patients undergoing a concomitant procedure, with however the definition of concomitant being within 10 days of randomization (ablation) and not necessarily at the time of the ablation procedure.

## Conclusion

Combined CA and LAAO is a cost-effective and beneficial treatment alternative for patients with symptomatic AF and high thromboembolic and bleeding risk, showcasing great procedural success and low periprocedural and follow-up complications. Nevertheless, the increased PDL rate observed in some studies need further investigation, in respect to their clinical significance. Larger scale studies are needed to verify the existing evidence on the beneficial role of the combined procedure and better define the optimal candidates.

## Data Availability

Not applicable.
